# Turning a *fasthug* [1] into *low flat hugs* could improve compliance with daily care bundles on the general intensive care unit: a preliminary audit

**DOI:** 10.1186/2197-425X-3-S1-A145

**Published:** 2015-10-01

**Authors:** M Brooke, B Murthy

**Affiliations:** University of Warwick, Medical Teaching Centre, Coventry, United Kingdom; University Hospital Coventry and Warwick, Dept of Anaesthesia, Coventry, United Kingdom

## Introduction

Over recent years, Intensive Care Medicine has recognised the benefit of implementing checklists to augment strategies for improving patient safety[2]. One such care bundle checklist is the FASTHUG mnemonic (Feeding; Analgesia; Sedation; Thromboprophylaxis; Head up positioning; Ulcer Protection and Glucose levels)[1]. In our ICU a variation of the mnemonic has evolved, initially into 'FLATHUGS', and more recently into 'FLATHUGS VC', where additional care bundle components include L: *invasive vascular****L****ines;* C: ***C****hlorhexidine mouth care*; V: *lung protective****V****entilation*.

## Objectives

This clinical audit examines (i) compliance with a locally adapted version of the FASTHUG[1] mnemonic, and (ii)suggests an updated version and template to improve its clinical utility.

## Methods

A random convenience sample of medical notes from 92 separate patient day reviews over a 4 week period were analysed for documented versions of FLATHUGS VC. Data was collected from all patients with an ICU stay >24 hours, and any actions raised during the documentation of FLATHUGS were checked for completion within that same 24 hour period.

## Results

In our unit, a basic 'FLATHUGS' package was completed in 81.5% of cases. The two most recently introduced components in addition to FLATHUGS (*lung protective ventilation;* and *Chlorhexidine mouth care)* were documented in 38% and 3.3% of cases respectively (Table 1). In addition, we found there were inconsistencies in the detail of what was recorded under each heading. Common examples included: (i) recording either the type of sedation, or the level of sedation (RASS Score); (ii) date of IV line *insertion* or anticipated date of IV line *replacement*.

## Conclusions

**Figure 1:**
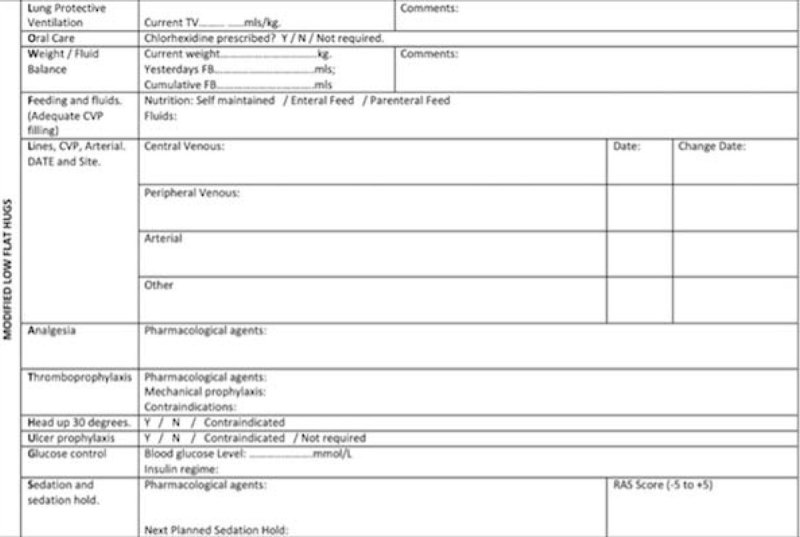
**LOW FLAT HUGS Template.**

There is currently an inconsistent approach amongst our ICU physicians in their application of the ´FASTHUGS VC´ care bundle, and we feel it is conceivable that this situation may be partially attributable to degradation of the original mnemonic. Although FASTHUG is an excellent mnemonic for a basic care bundle, we suggest it could be further improved by amending it to 'LOW FLAT HUGS', which includes four additional elements to those described by Vincent[1] in the original version (*Lung protective ventilation; Oral hygiene of intubated patients; Weight change; Line change with date*). To help embed these proposed changes and improve compliance a template sticker (Figure 1) may prove useful, and further evaluation after a period of implementation is recommended.

**Table 1:** Completion of FLATHUGS (and variant).

	Patient Day Reviews	Percentage Completion
Total	92	
´FLATHUGS´ completed	75	81.5%
´FLATHUGS´ attempted but not completed	14	15%
´FLATHUGS´ omitted	3	3.3%
´FLATHUGS V´	35	38%
´FLATHUGS VC´	3	3.3%
